# MosaicExplorerJ: Interactive stitching of terabyte-size tiled datasets from lightsheet microscopy

**DOI:** 10.12688/f1000research.27112.2

**Published:** 2021-02-04

**Authors:** Sébastien Tosi, Lídia Bardia, Maria Jose Barallobre, Arrate Muñoz-Barrutia, María Luisa Soto-Montenegro, Julien Colombelli

**Affiliations:** 1 Institute for Research in Biomedicine - IRB Barcelona, the Barcelona Institute for Science and Technology - BIST, Barcelona, Spain; 2 Department of Developmental Biology, Instituto de Biología Molecular de Barcelona, CSIC, Barcelona, Spain; 3 Departamento de Bioingeniería e Ingeniería Aeroespacial, Universidad Carlos III de Madrid, Leganés, Spain; 4 Instituto de Investigación Sanitaria Gregorio Marañón, Madrid, Spain; 5 CIBER de Salud Mental (CIBERSAM), Madrid, Spain

**Keywords:** 3D stitching, Lightsheet microscopy, Tiled scan, Mosaic, ImageJ

## Abstract

We introduce MosaicExplorerJ, an ImageJ macro to stitch 3D tiles from terabyte-size microscopy datasets organized on a regular 2D grid. As opposed to existing software, stitching does not require any prior information on the actual positions of the tiles, or conversion of raw TIFF images to a multi-resolution format for interactive exploration and fast processing. MosaicExplorerJ was specifically designed to process lightsheet microscopy datasets from optically cleared samples. It can handle multiple fluorescence channels, dual-sided lightsheet illumination and dual-sided camera detection.

## Introduction

**Figure 1.**
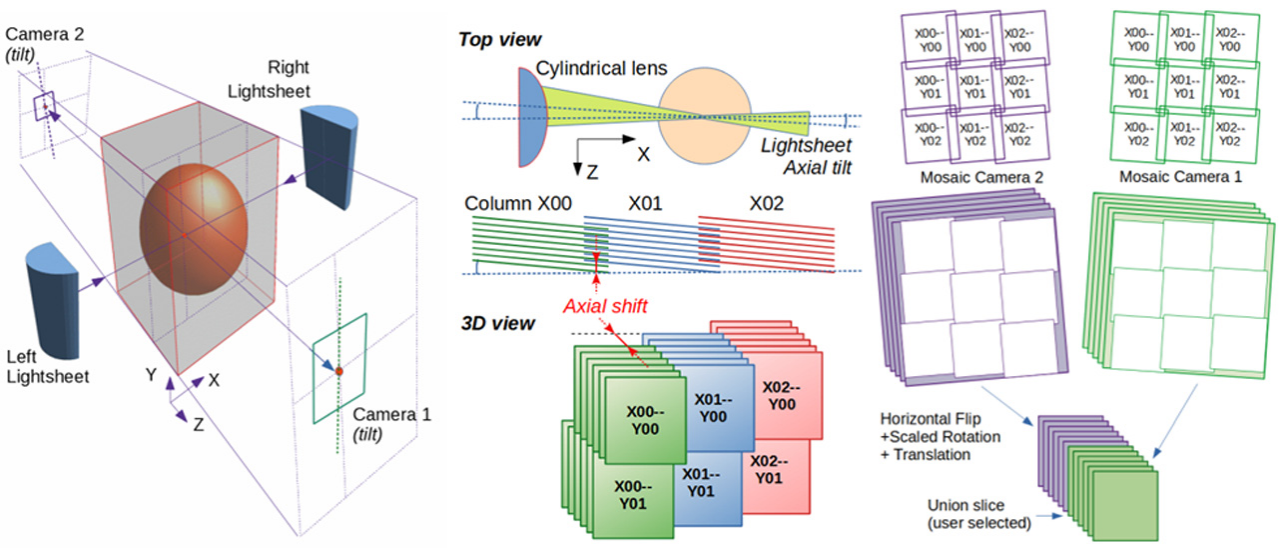
**Left**: Illuminating the sample and collecting the light from both sides enables to image a sample by lightsheet microscopy in the best conditions. **Middle**: Overlapping tiles (green, blue, red) from a 3D mosaic are shifted axially to compensate for the tilt of the lightsheet (here exaggerated around Y axis). **Right**: Reconstructed mosaics from both cameras are aligned before stacking their best section.

A number of open source tools are available to stitch mosaics from optical microscopy 3D tiled scans ^1– 4^ but they systematically implement automated algorithms which results might depend on the starting positions of the tiles and potentially converge to a suboptimal solution. This is especially likely if the initial positions are far from the optimal positions, or if the data suffers from unexpected artifacts. Even worse, this situation can be difficult to detect in practice since these tools often bring no or scarce support to check the results and finely correct for the observable residual errors manually. Additionally, some software limits the size of the datasets ^1, 2^, or requires the data to be redundantly converted to ad-hoc formats ^3^. Finally, until now only BigStitcher ^4^ could handle dual-sided illumination and dual-sided camera detection, two useful lightsheet microscopy ^5^ modalities that can advantageously be combined ( Figure 1, Left). We developed MosaicExplorerJ to address these shortcomings and bring a complementary alternative to ImageJ BigStitcher, the reference in the field.

## Methods

### Implementation

Whereas stitching the tiles of confocal microscopy datasets chiefly consists in compensating for the scanning head to sample stage tilt (assuming a perfectly orthogonal XYZ translation system), stitching lightsheet microscopy datasets is compounded by the fact that the lightsheet is not necessarily perfectly collinear to the object plane of the detection objective. This can lead to 1) a distortion of the aspect ratio of the images (often negligible), 2) an apparent axial displacement of the tiles while moving across the mosaic. This second effect can be simply compensated by axially offsetting the 3D tiles accordingly ( Figure 1, Middle), but additional lightsheet non-uniformity (or lateral misalignment) can lead to differences in the features visible in the regions of tile overlap; potentially weakening correlation based stitching algorithms. To address these issues, MosaicExplorerJ assists the user in visually aligning the tiles along the possible degrees of freedom set by a predefined physical model by following a step-by-step procedure to compensate for mismatches highlighted in the regions of tiles overlap.

### Operation

**Figure 2.**
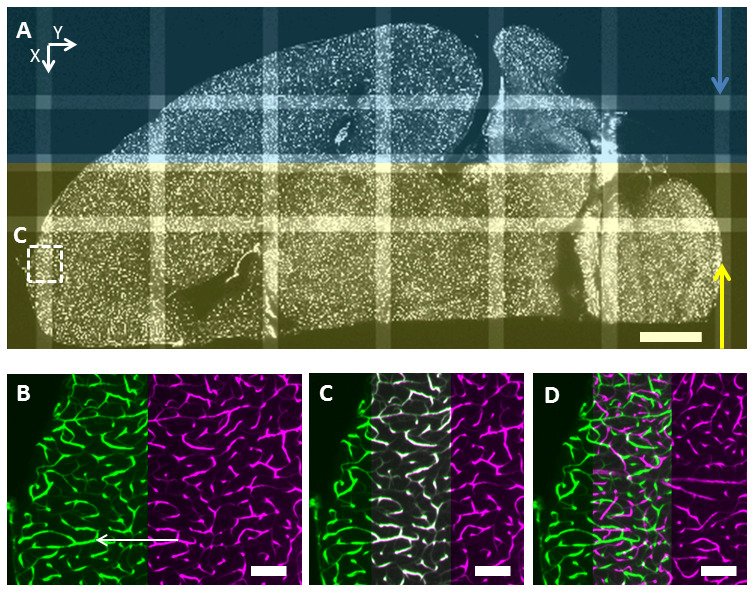
**A**: Two mosaics (2×8 tiles each with a full overlap in the central column along Y) from a dual-sided illumination dataset aligned with MosaicExplorerJ, the arrows show the directions of the light sheets (the view is turned and cropped to fit the figure). Scale bar: 1 mm. **B**: Zoomed region from the red square prior to adjusting the XY positions of the tiles but after axial shift (Z) correction. The user joins two matching features (white arrow) from two adjacent tiles. **C**: The XY positions of the tiles are adjusted based on the previous user input and for correct alignment matching features are highlighted in white in the region of overlap. **D**: Same view but prior to axial shift correction to compensate for lightsheet tilt: no matching features are apparent in the region of overlap. Scale bar: 100 µm.

ImageJ/Fiji should be installed and MosaicExplorerJ run from ImageJ macro editor. If no other data is available, the software can be tested with the sample data provided (Extended Data S1 ^6^). First, the mosaics from both cameras (and illumination sides) should be aligned independently before being aligned together. This first operation can be as simple as joining two matching features between two adjacent tiles ( Figure 2B, Extended Data S3-V1 ^6^), while compensating lightsheet tilt ( Figures 2C, 2D) and the axial wobbling of the motors forming the mosaics might require to adjust the axial shift of a selected number of tiles in the top row/column of the mosaic (Extended Data S3-V2 ^6^). Dual-sided camera datasets alignment includes an extra calibration step to compensate for discrepancy between the magnifications of both detection objective lenses ( Figure 1, Right, Extended Data S3-V3 ^6^), while dual-sided illumination datasets can be stitched by following a similar procedure (Extended Data S3-V4 ^6^). Intensity correction can be achieved from either external correction masks or interactively adjusted from XY separable shading correction masks (Extended Data S3-V5 ^6^). Finally, the user can save the overall mosaic alignment for further inspection, or export the stitched dataset as a TIFF series.

### Testing

MosaicExplorerJ has been extensively tested by stitching several large lightsheet microscopy datasets acquired from diverse optically cleared biological samples (see *Data availability*).

## Use cases

All datasets were successfully aligned, each time in under 30 minutes. The results were checked visually by scrolling through the slices and ensuring that the alignment was correct in the regions of overlap between the tiles. The dataset Brain2_izq_2x8Mosaic_LeftSide_300GB was also aligned by BigStitcher, leading to similar results both visually and quantitatively (Extended Data Table S1 ^6^). It took about 2h30 to process this dataset with BigStitcher (from 3D TIF tiles to computed alignment), excluding the conversion from TIFF series (the original format of this dataset) to 3D TIFF tiles. This time is expected to scale at least linearly with increasing dataset size for BigStitcher while alignment time in MosaicExplorerJ is relatively constant, and mostly conditioned by the degrees of freedom of the 3D tiles. After alignment, the exportation of the stitched images to TIFF series could be achieved in a comparable time with both tools.

## Conclusion

Even though it only supports a subset of its features, MosaicExplorerJ brings a complementary alternative to BigStitcher and presents a number of advantages (Extended Data S2 ^6^ and Table S2 ^6^). No fiducials or detectable feature points are required, which makes the tool robust, versatile, and compatible with large optically cleared samples for which introducing fiducials is virtually impossible. The processing is fast, and terabyte-size datasets can be explored on the fly without conversion to an intermediate format, even on laptop computers with limited memory. Finally, all alignment steps are performed visually, which brings direct control and feedback both on the imperfections of the datasets and on the quality of the results, minimizing the risk of leaving coarse errors unnoticed.

## Data availability

### Underlying data

A complete description of the datasets used to test MosaicExplorerJ, including sample preparation and imaging can be found in Extended Data S1 ^6^. The full datasets are too large (1.5TB) to feasibly host on a data repository; however, datasets can all be accessed publicly on the IRB Barcelona Google Drive at: https://bit.ly/37iocrP.

### Extended data

Zenodo: MosaicExplorerJ F1000Research article extended data. 10.5281/zenodo.4455761 ^6^. **

Data are available under the terms of the Creative Commons Attribution 4.0 International license (CC-BY 4.0).

## Software availability

Source code and documentation are available from: https://github.com/SebastienTs/MosaicExplorerJ

Archived source code at time of publication: http://www.doi.org/10.5281/zenodo.4453506 ^7^.

License: GNU General Public License v3.0.
