# Supplementary material for: MosaicExplorerJ: Interactive stitching of terabyte-size tiled datasets from lightsheet microscopy
Source: F1000Res. 2021 Feb 4;9:1308. Originally published 2020 Nov 10. [Version 2] doi: 10.12688/f1000research.27112.2 (PMC7953913; doi:10.12688/f1000research.27112.2)
Supplement: Peer review history

# Peer review: Reviewer response for version 2

**DOI:** 10.5256/f1000research.54151.r78912

**Published:** 2021-03-11

**Authors:** Stephan Preibisch

**Affiliations:** 1 Berlin Institute for Medical Systems Biology, Berlin, Germany; 2 HHMI Janelia, Ashburn, VA, USA

Thanks so much to the authors for addressing my concerns, I think their work is a useful addition for lightsheet reconstruction.

Are the conclusions about the tool and its performance adequately supported by the findings presented in the article?

Partly

Is the rationale for developing the new software tool clearly explained?

Partly

Is the description of the software tool technically sound?

Partly

Are sufficient details of the code, methods and analysis (if applicable) provided to allow replication of the software development and its use by others?

Yes

Is sufficient information provided to allow interpretation of the expected output datasets and any results generated using the tool?

Yes

Reviewer Expertise:

Image analysis, image registration, lightsheet

I confirm that I have read this submission and believe that I have an appropriate level of expertise to confirm that it is of an acceptable scientific standard.

# Peer review: Reviewer response for version 2

**DOI:** 10.5256/f1000research.54151.r78911

**Published:** 2021-03-01

**Authors:** Francesco Pampaloni

**Affiliations:** 1 Buchmann Institute for Molecular Life Sciences, Goethe University Frankfurt, Frankfurt am Main, Germany

The authors added links to five video tutorials in the documentation available on GitHub. The videos greatly support the first user of the MosaicExplorerJ macro in understanding the different steps to align and merge the tiles and to perform the registration of dual-sided LS illumination and dual-sided detection data sets. The changes and the addition in the documentation made by the authors in the reviewed version significantly reduce the time the user has to invest to learn how to apply the macro to her/his own data sets. Especially after following the explanatory videos, all the functions of the macro can be straightforwardly used. Overall, a user-friendly and very useful macro for the light sheet microscopy community.

 Detected bugs:

- After pressing Cancel, it is not possible to re-open the MosaicExplorerJ GUI with Alt.
- The “Register OvIX & YCor -> OvIY &XCor” function works correctly only if the selection line is drawn from east -> west and not vice versa.
- The “Register OvY  XCor only” function works correctly only if the selection line is drawn from the bottom to the top (or south -> north), but not vice versa.

Are the conclusions about the tool and its performance adequately supported by the findings presented in the article?

Yes

Is the rationale for developing the new software tool clearly explained?

Yes

Is the description of the software tool technically sound?

Yes

Are sufficient details of the code, methods and analysis (if applicable) provided to allow replication of the software development and its use by others?

Partly

Is sufficient information provided to allow interpretation of the expected output datasets and any results generated using the tool?

Yes

Reviewer Expertise:

Light Sheet Microscopy, 3D cell biology, stem cell and tumor organoids.

I confirm that I have read this submission and believe that I have an appropriate level of expertise to confirm that it is of an acceptable scientific standard.

# Peer review: Reviewer response for version 1

**DOI:** 10.5256/f1000research.29948.r75056

**Published:** 2020-12-11

**Authors:** Francesco Pampaloni

**Affiliations:** 1 Buchmann Institute for Molecular Life Sciences, Goethe University Frankfurt, Frankfurt am Main, Germany

Overview

 The MosaicExplorerJ ImageJ macro-code described in the manuscript is a useful tool to stitch very large data sets recorded with light sheet microscopy. Indeed, the amount of data acquired in a typical session of light-sheet imaging of optically cleared organs (e.g. the mouse brain), reaches easily the TB range with up-to-date equipment. Therefore, user-friendly and straightforward data navigation and stitching software is needed by the scientific community. MosaicExplorerJ, at variance with automatic stitching algorithms such as BigStitcher, it is a fully manual tool, requiring the user manually perform all the alignment steps in order to compensate the tiles mismatch, e.g. the tiles axial shift arising from a tilted light sheet. Moreover, also at variance with BigStitcher, multi-tiff image stacks can be opened directly without conversion.

 I recommend the manuscript for idexing with minor changes and additions as described in the following remarks.

Remarks

 The dataset deposited by the authors on the IRB Barcelona server allows readily testing the macro. The macro runs from the macro editor. The dataset is quickly loaded into the main window. In order to properly upload the tiles, the different files have to be named sequentially following the explained rule. After opening the dataset, the alignment and stitching are performed by manually setting the shifting parameters (xy translation and axial alignment along z) shown in the control panel.

 In general, a first-time user can achieve the stitching of the mosaic 3D dataset as described in the manuscript by following the steps outlined in the documentation available on GitHub. However, the learning curve is quite steep and some effort and time is necessary by the user in order to understand the working of the macro and to achieve the result. In order to allow for a smoother learning curve, I strongly recommend the authors to upload videos illustrating the stitching procedure for two datasets at increasing difficulty.

 Particularly, the following step described in the documentation (p. 3, end) is not clearly explained, and should be better illustrated with a further figure and in a video:

*“For linear correction, it is recommended to use the top left tile as reference and gradually tweak ZxCor from the tile to the right, until some matching features are apparent in the overlap region. Then ZyCor can be adjusted, this time by considering the tile below. The adjustment of the Z offsets can be performed by typing values in the Panel or by following the procedure described below for free correction.”*

 I also recommend to include the documentation in the manuscript extended material as well, and not exclusively in GitHub, as the macro “instructions” are an essential part of the work.

Are the conclusions about the tool and its performance adequately supported by the findings presented in the article?

Yes

Is the rationale for developing the new software tool clearly explained?

Yes

Is the description of the software tool technically sound?

Yes

Are sufficient details of the code, methods and analysis (if applicable) provided to allow replication of the software development and its use by others?

Partly

Is sufficient information provided to allow interpretation of the expected output datasets and any results generated using the tool?

Yes

Reviewer Expertise:

Light Sheet Microscopy, 3D cell biology, stem cell and tumor organoids.

I confirm that I have read this submission and believe that I have an appropriate level of expertise to confirm that it is of an acceptable scientific standard, however I have significant reservations, as outlined above.

# Response

**Published:** 2021-01-21

**Authors:** Sebastien Tosi

Thank you for reviewing the article and for pointing out these weaknesses of the documentation. We have tried to substantially improve the documentation accordingly and we have created five video tutorials to help illustrate all the steps described in the documentation. Please see our detailed answers below.

 > In general, a first-time user can achieve the stitching of the mosaic 3D dataset as described in the manuscript by following the steps outlined in the documentation available on GitHub. However, the learning curve is quite steep and some effort and time is necessary by the user in order to understand the working of the macro and to achieve the result. In order to allow for a smoother learning curve, I strongly recommend the authors to upload videos illustrating the stitching procedure for two datasets at increasing difficulty.

 We have included five video tutorials that illustrate the main operations and complement the documentation. These videos are available from the GitHub repository and from the new Extended Data **S3**.

 > Particularly, the following step described in the documentation (p. 3, end) is not clearly explained, and should be better illustrated with a further figure and in a video: “For linear correction, it is recommended to use the top left tile as reference and gradually tweak ZxCor from the tile to the right, until some matching features are apparent in the overlap region. Then ZyCor can be adjusted, this time by considering the tile below. The adjustment of the Z offsets can be performed by typing values in the Panel or by following the procedure described below for free correction.”

 We have completely rewritten this part of the documentation and the new video tutorial **S3-V2** is fully dedicated to explaining this critical step.

 > I also recommend to include the documentation in the manuscript extended material as well, and not exclusively in GitHub, as the macro “instructions” are an essential part of the work.

 We created a new Extended Data **S3** with links to the documentation of the software and to the five new video tutorials. Both are also available from the GitHub repository.

# Peer review: Reviewer response for version 1

**DOI:** 10.5256/f1000research.29948.r74555

**Published:** 2020-12-07

**Authors:** Stephan Preibisch

**Affiliations:** 1 Berlin Institute for Medical Systems Biology, Berlin, Germany; 2 HHMI Janelia, Ashburn, VA, USA

The authors present MosaicExplorerJ, an ImageJ macro for manual alignment of tiled lightsheet acquisitions.

 I think it can be useful as it provides simple, editable source code. I personally found it quite hard to use, but more practice might make it more efficient. But more explanations of the parameters and keyboard shortcuts in the application would certainly be helpful (press F1 or similar).

 Most importantly, the paper should clearly state the limitations of the software and correct wrong statements about the comparison to other tools, in particular BigStitcher from my point of view.

Not mentioned limitations (or implicitly assumed ones):

- all files need to have the same dimensions (also in z)
- only 3D translation models are supported
- no chromatic aberration correction
- maximal xy output size is 2^31 pixels, the limitation of ImageJ (around 47000x47000 pixels)

Incorrect comparisons that should be corrected:

 1) “As opposed to existing software, stitching does not require any prior information on the actual positions of the tiles, sample fiducials, or conversion of raw TIFF images, and the stitched images can be explored instantly.”

- That is the same in BigStitcher, conversion to HDF5 is not necessary, TIFFs can be virtually loaded and displayed instantaneously 

2) “but they all implement automated algorithms … Even worse, this situation can be difficult to detect in practice since these tools bring no or scarce support to check the results and correct for errors manually.”

- BigStitcher provides user friendly methods for manual & interactive alignment (3D translation): https://imagej.net/BigStitcher_manual_translation

3) “Finally, none of these solutions handles both dual-side illumination and dual-side camera detection, two useful lightsheet microscopy”

- BigStitcher supports dual-side illumination and dual-side camera detection (e.g. Supplementary Figure 17)

4) S2-Comparison with BigStitcher:

- 3D TIFF tiles can be natively used through virtual loading, live display in BDV even. HDF5 conversion is optional (yet very useful)
- 2D TIFF slices are also supported, but it's a quite hidden feature]
- BigStitcher supports Dual-camera support
- Physical model constrained grid alignment is also possible, see link above, even interactive & manual
- Automatic color highlighting of tiles mismatch can be done in BigStitcher
- BigStitcher additionally supports per-tile intensity correction using Affine 1D models

Are the conclusions about the tool and its performance adequately supported by the findings presented in the article?

Partly

Is the rationale for developing the new software tool clearly explained?

Partly

Is the description of the software tool technically sound?

Partly

Are sufficient details of the code, methods and analysis (if applicable) provided to allow replication of the software development and its use by others?

Yes

Is sufficient information provided to allow interpretation of the expected output datasets and any results generated using the tool?

Yes

Reviewer Expertise:

Image analysis, image registration, lightsheet

I confirm that I have read this submission and believe that I have an appropriate level of expertise to confirm that it is of an acceptable scientific standard, however I have significant reservations, as outlined above.

# Response

**Published:** 2021-01-21

**Authors:** Sebastien Tosi

First of all, we thank the reviewer for his thorough comments and we would like to apologize for overlooking some of BigStitcher's features that we were genuinely unaware of. Also, as rightly pointed out by the reviewer, some limitations of MosaicExplorerJ were not discussed in the article. We have remedied this, as described point by point in our answer, and we hope that the features and the operating workflow of the two applications are now reflected more accurately in the fully updated Extended Data **S2**.

 > More explanations of the parameters and keyboard shortcuts in the application would certainly be helpful (press F1 or similar).

 We have tried our best to improve this aspect in MosaicExplorerJ (v1.5, now on GitHub): a help window can now be displayed by pressing F1, contextual information is provided in ImageJ status bar (and strategically in some of the dialog boxes), and the manual operation to perform axial adjustment of the tiles has been simplified. Finally, five video tutorials ( bit.ly/2KY0BDN) were created to illustrate the main operations and to complement the documentation.

 > Not mentioned limitations (or implicitly assumed ones) of MosaicExplorerJ:

 1. All files need to have the same dimensions (also in z)Actually, this is not strictly required as missing tiles or tiles missing Z slices (for 2D TIFF series mode) will not trigger an error but the corresponding images will just be displayed black. However, to answer this comment, we now state in the Abstract of the article (and in Extended Data **S2**) that the tiles should be organized on a regular 2D grid.

 2. Only 3D translation models are supported

 Yes, only these operations are supported for a single camera dataset but 2D similarity (rotation, scaling, translation) is performed (after 3D translations) when registering the mosaics from two opposing cameras (as described in **Figure 1**).

 3. No chromatic aberration correction

 We now explicitly state in Extended Data **Table S2 **that chromatic aberrations correction is not supported by MosaicExplorerJ (while it is supported by BigStitcher).

 4. Maximal xy output size is 2^31 pixels, the limitation of ImageJ (around 47000x47000 pixels)

 We now explicitly state in Extended Data **Table S2 **that mosaics stitched by MosaicExplorerJ are limited to about 47.000 x 47.000 pixels (and eight channels) while BigStitcher sets no limit. We haven’t included this information in the text of the main article as we believe that it is not really limiting in practice.

Incorrect comparisons that should be corrected:

 1) “As opposed to existing software, stitching does not require any prior information on the actual positions of the tiles, sample fiducials, or conversion of raw TIFF images, and the stitched images can be explored instantly.”

 That is the same in BigStitcher, conversion to HDF5 is not necessary, TIFFs can be virtually loaded and displayed instantaneously.

 We apologize for this misleading statement and we were unaware that tiles could be loaded as a set of 2D TIFF series in BigStitcher. Still, we believe that HDF5 conversion is implicitly required for efficient operations when handling TB datasets, both for interactive 3D viewing and to enable a practical processing time (especially when several rounds of tile placement / parameters optimizations have to be performed to get to satisfying results). We updated this paragraph of the text to reflect this idea, and we stated in Extended Data **Table S2** that while not strictly required HDF5 conversion is desirable to speed up operations.

 2) “but they all implement automated algorithms … Even worse, this situation can be difficult to detect in practice since these tools bring no or scarce support to check the results and correct for errors manually.”

 BigStitcher provides user friendly methods for manual & interactive alignment (3D translation): https://imagej.net/BigStitcher_manual_translation

 We were aware of this feature but, as far as we can tell, tiles are first automatically laid down to a regular grid (with fixed overlap), and then only individual tiles (or whole groups) can be interactively moved. This feature is certainly useful to coarsely set the initial positions of the tiles but not so much to finely adjust the mosaic (that would ultimately require to finely move tiles on a tile by tile basis). In turn, thanks to the predefined physical correction models, as few as two pairs of tiles need to be manually adjusted in MosaicExplorerJ to stitch a whole mosaic (with no further processing). We tried to reflect this more accurately in this paragraph and in Extended Data **Table S2**.

 3) “Finally, none of these solutions handles both dual-side illumination and dual-side camera detection, two useful lightsheet microscopy”

 BigStitcher supports dual-side illumination and dual-side camera detection (e.g. Supplementary Figure 17)

 We apologize for this plainly wrong statement (we did not know that BigStitcher supported dual-sided cameras as the option is not listed in the image loader). We modified the text to exclude BigStitcher from the set of software not supporting the combination of these two modalities and we reflected these features in details in Extended Data **S2 **and Extended Data **Table S2**.

 4) S2-Comparison with BigStitcher:

 3D TIFF tiles can be natively used through virtual loading, live display in BDV even. HDF5 conversion is optional (yet very useful)

 2D TIFF slices are also supported, but it's a quite hidden feature

 Extended Data **Table S2** has been updated to reflect this.

 BigStitcher supports Dual-camera support

 Extended Data **Table S2** has been updated to reflect this.

 Physical model constrained grid alignment is also possible, see link above, even interactive & manual

 For the mosaic stitching part only (excluding cameras registration), MosaicExplorerJ physical correction models include camera tilt (XY translations), lighshsheet tilt (linearly increasing Z translations) and motor axis axial wobble (XY separable Z translations). BigStitcher global tile layout only supports a regular grid with fixed overlap (not accounting for previously mentioned effects). In our view, this is useful to coarsely set the starting positions of the tiles prior to optimization but it is really practical to finely adjust the positions of the tiles of large mosaics with no further processing. We tried to reflect this more accurately in the text and in Extended Data **S2**.

 Automatic color highlighting of tiles mismatch can be done in BigStitcher

 Extended Data **Table S2** has been updated to reflect this. MosaicExplorerJ supports alternate red/green (and cyan/yellow) while BigStitcher supports random colors.

 BigStitcher additionally supports per-tile intensity correction using Affine 1D models

 Extended Data **Table S2** has been updated to reflect this.
